# Amentoflavone promotes ferroptosis by regulating reactive oxygen species (ROS) /5’AMP-activated protein kinase (AMPK)/mammalian target of rapamycin (mTOR) to inhibit the malignant progression of endometrial carcinoma cells

**DOI:** 10.1080/21655979.2022.2079256

**Published:** 2022-05-29

**Authors:** Qi Sun, Peng Zhen, Dandan Li, Xiaochen Liu, Xinling Ding, Huihui Liu

**Affiliations:** a Traditional Chinese Medicine (Mongolian Medicine) College, Chifeng University, Chifeng, Inner Mongolia, China; b Department of Radiation Oncology, Chifeng Cancer Hospital, Chifeng, Inner Mongolia, China; c Rehabilitation Department, Chifeng Cancer Hospital, Chifeng, Inner Mongolia, China; d Department of Human Anatomy, Basic Medical College, Chifeng University, Chifeng, Inner Mongolia, China; e Cancer Rehabilitation Department, Chifeng Cancer Hospital, Chifeng, Inner Mongolia, China

**Keywords:** Amentoflavone, ferroptosis, ROS/AMPK/mTOR, endometrial cancer cells

## Abstract

It was reported that amentoflavone (AF) had anti-tumor ability. Therefore, this study aimed to investigate the role of AF in endometrial cancer as well as to discuss its underlying mechanism. The viability, proliferation, and apoptosis of endometrial carcinoma cells (KLE) with AF administration were detected by methyl tetrazolium (MTT) assay, clone formation, and terminal deoxynucleotidyl transferase (TdT) dUTP Nick-End Labeling (TUNEL) assays. Thiobarbituric acid reactive substance (TBARS) production and Fe^2+^ level in AF-treated KLE cells were detected by TBARS assay and Iron assay. The expressions of proliferation- apoptosis-, ferroptosis-, and 5'AMP-activated protein kinase (AMPK)/mammalian target of rapamycin (mTOR) signaling-related proteins in AF-treated KLE cells were detected by western blot analysis. Reactive oxygen species (ROS) expression in AF-treated KLE cells was determined by ROS assay kit. N-acetyl cysteine (NAC), which is an inhibitor of ROS, was used to confirm whether AF exerted its effects on KLE cells through ROS/AMPK/mTOR signaling. As a result, AF inhibited the viability and proliferation of KLE cells but promoted apoptosis and ferroptosis. The expressions of ROS and AMPK were increased, while mTOR expression was decreased in AF-treated KLE cells. NAC reversed the effects of AF on biological behaviors of KLE cells by inactivating ROS/AMPK/mTOR signaling. In conclusion, AF promoted ferroptosis by activating ROS/AMPK/mTOR to inhibit the viability and proliferation and promoted the apoptosis and ferroptosis of KLE cells.

## Graphical abstract

**Figure:**
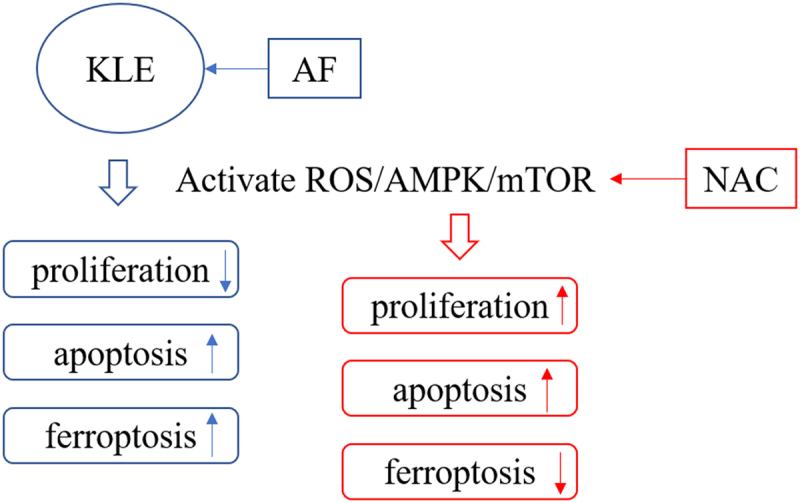

## Highlights

- AF inhibits the proliferation and promotes apoptosis of endometrial cancer cells.
- AF promotes ferroptosis in endometrial cancer cells.
- AF suppresses ROS/AMPK/mTOR signaling pathway.
- The effect of AF on endometrial cancer cells is reversed by NAC (ROS inhibitor).

## Introduction

Endometrial cancer is a common gynecological malignancy, with an increasing incidence year by year and a trend of younger age [1,2]. Clinical epidemiological data showed that the progressed treatment methods have not significantly improved the overall survival of patients with endometrial cancer; in this way, endometrial cancer is still a major risk factor for women’s health [3]. The search for active antitumor components of Traditional Chinese medicine and the elucidation of its antitumor mechanism have become a research hotspot in this field.

As a polyphenolic compound, amentoflavone (AF) was extracted from selaginella and had been testified to have many biological properties, including anti-inflammatory, anti-oxidative, and anti-tumor properties [4–7]. AF-induced cell cycle arrest and apoptosis could inhibit cell proliferation, which depended on mitochondrial pathway of breast cancer cells in vitro [8]. AF inhibited ovarian cell proliferation, interrupted microtubule dynamic balance, and stagnated cells in G2 phase [9]. However, the role of AF in the malignant progression of endometrial cancer has not been reported.

Low glucose activated 5'AMP-activated protein kinase (AMPK) signaling, induced cell cycle G1 arrest, and led to apoptosis of Ishikawa and ECC-1 endometrial cancer cells [10]. Artesunate induced apoptosis and cell cycle arrest of endometrial cancer cells by down-regulating ER-α expression and activating liver kinase B1 (LKB1)/AMPK/mammalian target of rapamycin (mTOR) pathways [11]. In ovarian cancer cells, AF regulated Skp2 through ROS/AMPK/mTOR pathway to exert antitumor effects [12]. Therefore, we speculated that AF might regulate endometrial cancer cells through AMPK signaling. Furthermore, dihydroartemisinin (DHA) effectively induced the ferroptosis of acute myeloid leukemia (AML) cells through autophagy by regulating the activity of AMPK/mTOR/p70S6k signaling pathway [13]. AF triggered ferroptosis in an autophagy-dependent manner, leading to decreased glioma cell viability and increased cell death [14]. Therefore, we speculated that AF might induce ferroptosis to regulate endometrial cancer cells through AMPK signaling.

Here, this study aimed to investigate whether AF promoted ferroptosis by regulating ROS/AMPK/mTOR to inhibit the viability and proliferation and promoted the apoptosis of endometrial cancer cells.

## Materials and methods

### Cell culture and treatment

Embryonic stem cell line (ESC) and endometrial carcinoma cell line (KLE) were all provided by the American Type Culture Collection (ATCC). ESC cells were cultured in 1:1 Mixture of Dulbecco’s Modified Eagles Medium and Ham’s F-12 medium (ATCC) supplemented with 2.0 mM L-Alanyl-L-Glutamine, 0.1 mM non-essential amino acids, 0.1 mM 2-mercaptoethanol, and 4 ng/ml bFGF as well as 15% fetal bovine serum (FBS). KLE cells were cultured in F12 Medium (ATCC) which was added with 10% FBS. All cells were incubated in 95% air and 5% CO_2_ at 37°C.

AF (purity >98.0%) and N-acetyl cysteine (NAC, ROS inhibitor) were all purchased from Sigma-Aldrich (USA). ESC cells and KLE cells were treated with AF at different concentrations (50, 75, and 100 μM) for 48 h [15].

In another experiment, KLE cells were pre-treated with NAC (2.5 mM) for 30 min [16] and then treated with 100 μM AF for 48 h.

### Methyl tetrazolium (MTT) assay

After the indicated treatment, KLE cells in 24-well plates were incubated at 37°C for 24 h. Each well was added with 1 ml of MTT solution (5 mg/ml) (Beyotime), and cells were incubated for 3 h at room temperature. Then, each well was added with 420 μl of DMSO, and then the plates were shaken for 15 min. The OD value was obtained at 490 nm by a microplate reader (Bio-Tek Instruments, Inc.) [17].

### Clone formation assay

After the indicated treatment, KLE cells were then cultured in DMEM: F12 Medium at 37°C for 2 weeks. After that, KLE cells were fixed with 4% paraformaldehyde (Beyotime) for 15 min and stained with 0.1% crystal violet (Beyotime) for 15 min. The colonies of KLE cells were observed and photographed with an Olympus digital camera (Olympus Corporation).

### Terminal deoxynucleotidyl transferase (TdT) dUTP Nick-End Labeling (TUNEL) assay

After the indicated treatment, KLE cells were fixed with 4% paraformaldehyde for 30 min at 4°C and then were permeabilized with 0.2% Triton X-100 (Beyotime) for 10 min at room temperature. The nuclei were counterstained with 0.5 µg/ml DAPI (Beyotime) at room temperature for 10 min. Finally, TUNEL-positive cells were viewed using a fluorescence microscopy (Olympus Corporation) [18].

### Western blot

After the indicated treatment, KLE cells were lysed with 0.5 μL RIPA lysis buffer (Beyotime) on ice for 30 min. Then, protein concentration was detected using a BCA protein assay kit. Subsequently, the proteins were separated by SDS-PAGE and then transferred onto polyvinylidene fluoride (PVDF) membranes (EMD Millipore). After being blocked with 5% nonfat milk for 2 hours at room temperature, membranes were incubated with primary antibodies against Ki67 (ab92742; dilution, 1:5000; Abcam), PCNA (ab92552; dilution, 1:5000; Abcam), Bcl-2 (ab32124; dilution, 1:1000; Abcam), Bax (ab182733; dilution, 1:2000; Abcam), cleaved-caspase3 (#9661; dilution, 1:1000; Cell signaling pathway), cleaved-caspase9 (Cat No: 10,380-1-AP; dilution, 1:1000; Proteintech), SLC7A11 (ab175186; dilution, 1:1000; Abcam), GPX4 (ab125066; dilution, 1:1000; Abcam), FTH1 (ab75972; dilution, 1:1000; Abcam), ACSL4 (Cat No: 22,401-1-AP; dilution, 1:2000; Proteintech), p-AMPK (ab92701; dilution, 1:1000; Abcam), p-mTOR (ab109268; dilution, 1:1000; Abcam), AMPK (ab32047; dilution, 1:1000; Abcam), mTOR (ab32028; dilution, 1:1000; Abcam) and GAPDH (ab9485; dilution, 1:2500; Abcam) overnight at 4°C. Subsequently, the membranes were washed with TBST for three times and then incubated with an appropriate HRP-conjugated secondary antibody for 2 h at room temperature. Protein bands were visualized using enhanced chemiluminescence reagent (Beyotime) and quantified using the Image J 1.8.0 software (National Institutes of Health).

### Thiobarbituric acid reactive substance (TBARS) assay

TBARS Assay Kit (Elabscience) was used to estimate lipid peroxidation in KLE cells after indicated treatment. Briefly, KLE cells were exposed to 0.5 ml 15% trichloroacetic acid and 10 μl of 500 mM butylated hydroxyanisole (BHA), after that centrifuged at 10,000 g for 10 min at 4°C. The obtained supernatant was added with 0.5 ml 0.375% thiobarbituric acid and then boiled for 10 min. After cooling, TBARS in the mixture was measured at 532 nm using a microplate reader (Bio-Tek Instruments, Inc.).

### Iron assay

Iron Assay Kit (Elabscience) was used to determine the concentration of Fe^2+^ in KLE cells after indicated treatment. Briefly, KLE cells were homogenized with iron assay buffer on ice and then centrifuged at 16,000 × g for 15 min at 4°C to collect lysis supernatant. Finally, a 100 μl lysis sample was incubated with 5 μl iron reducer for 30 min at 37 °C, followed by the addition of 100 μl iron probe. The mixture was incubated in the dark for 30 min at 37 °C. The absorbance was measured at 593 nm by a microplate reader (Bio-Tek Instruments, Inc.).

### Reactive oxygen species (ROS) assay

After the indicated treatment, KLE cells were treated with 10 μM DCFH-DA (1:1000) (Elabscience) and incubated at 37°C for 24 h. The KLE cells were washed with serum-free cell culture medium for three times. Images of ROS level were photographed under fluorescence microscopy (Olympus Corporation).

### Statistical analysis

Experimental data that collected from at least three independent experiments were presented as mean ± SD. All data were detected by Shapiro–Wilk (S-W) to evaluate whether they fit the normal distribution. If the data conformed to normal distribution, comparisons among multiple groups were conducted using one-way ANOVA followed by Tukey’s *post hoc* test. Statistical significance was identified at P < 0.05. Statistical analysis was performed using GraphPad Prism 8.0 (GraphPad Software, Inc.).

## Results

### Effects of AF on the viability of endometrial cancer cells

**Figure 1.**
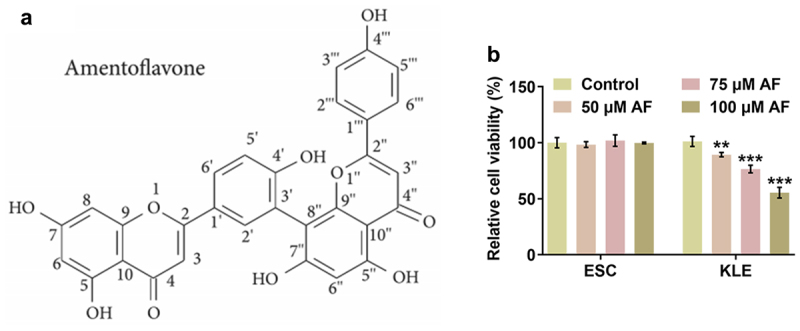
Effects of AF on the viability of endometrial cancer cells. (a) The chemical structure of AF. (b) The viability of ESC cells and KLE cells treated by different concentrations of AF was detected by CCK-8 assay. **P < 0.01 and ***P < 0.001 vs. Control group.

To investigate the effects of AF on the viability of ESC cells and KLE cells, MTT assays were applied. The chemical structure of AF is shown in Figure 1a. When ESC cells and KLE cells were treated with different concentrations of AF (50, 75, and 100 μM), the viability of ESC cells was not significantly affected but the viability of KLE cells was gradually decreased in a dose-dependent manner (Figure 1b).

### AF inhibited the proliferation and promoted the apoptosis of endometrial cancer cells

**Figure 2.**
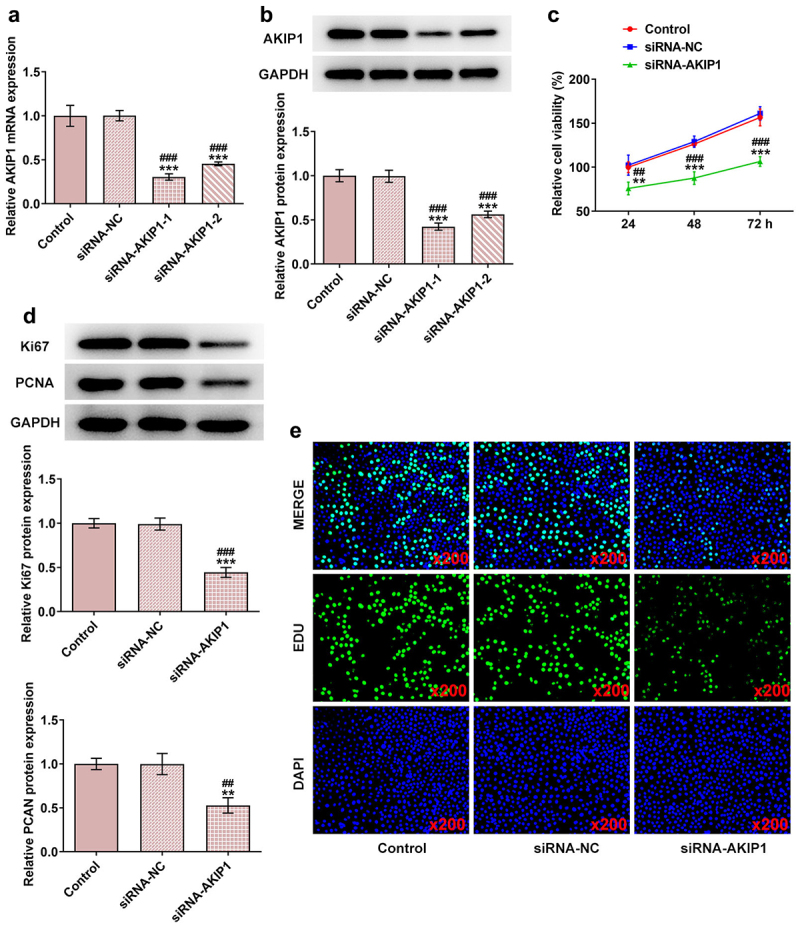
AF inhibited the proliferation and promoted the apoptosis of endometrial cancer cells. The viability (a) and proliferation (b) of KLE cells treated by different concentrations of AF were detected by CCK-8 and clone formation assays. (c and d) The apoptosis of KLE cells treated by different concentrations of AF was analyzed by TUNEL assay. (e) The expression of proteins associated with proliferation and apoptosis in KLE cells treated by different concentrations of AF was determined by western blot. *P < 0.05, **P < 0.01 and ***P < 0.001 vs. Control group.

The proliferation and apoptosis of AF-administrated KLE cells were detected by MTT, colony formation, and TUNEL assays. The viability and proliferation of KLE cells were inhibited by AF in a dose-dependent manner (Figure 2a and b). AF promoted the apoptosis of KLE cells in a concentration-dependent manner (Figure 2c and d). The expressions of proteins associated with proliferation and apoptosis were changed as follows: the expressions of Ki67, PCNA, and Bcl-2 showed a gradual decline, and the expressions of Bax, cleaved-caspase3/caspase3 and cleaved-caspase9/caspase9 had gradually risen in KLE cells treated by AF with varying concentrations (50, 70, and 100 μM) (Figure 2e).

### AF promoted ferroptosis in endometrial cancer cells

**Figure 3.**
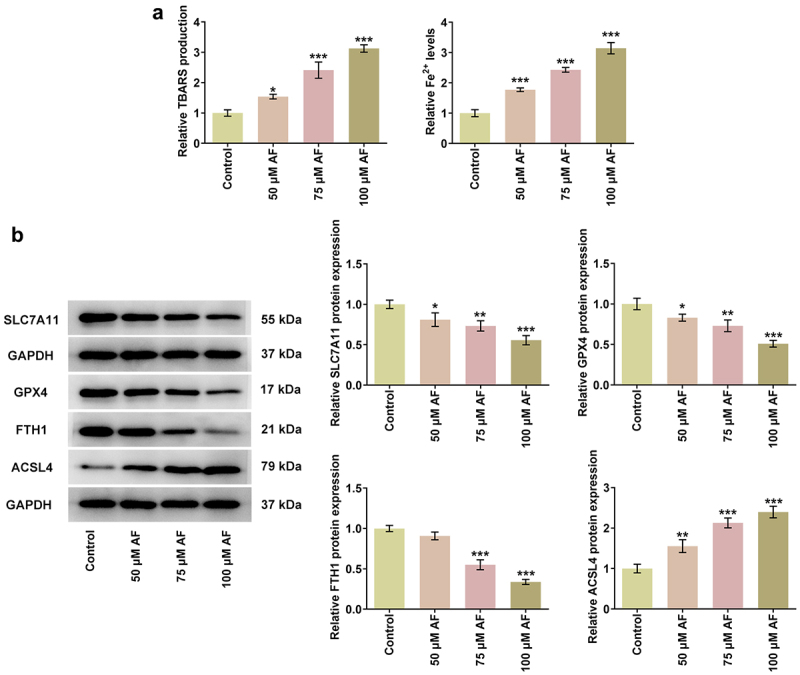
AF promoted ferroptosis in endometrial cancer cells. The TBARS production (a) and level of Fe^2+^ (b) in KLE cells treated by different concentrations of AF were detected by TBARS and Iron assays. (c) The expressions of proteins associated with ferroptosis in KLE cells treated by different concentrations of AF was determined by western blot. *P < 0.05, **P < 0.01 and ***P < 0.001 vs. Control group.

The ferroptosis of AF-treated KLE cells was analyzed. TBARS production and the level of Fe^2+^ all showed an increased trend with AF administration (Figure 3a and 3b). The measurement of ferroptosis-related proteins in KLE cells showed that the expressions of SLC7A11, GPX4, and FTH1 were gradually decreased, while the expression of ACSL4 was gradually increased by AF in a dose-dependent manner (Figure 3c).

### AF regulated ROS/AMPK/mTOR signaling pathway

**Figure 4.**
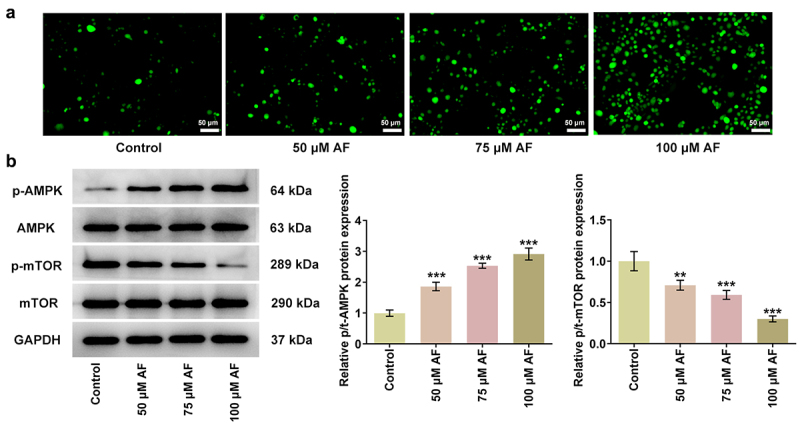
AF regulated ROS/AMPK/mTOR signaling pathways. (a) The ROS expression in KLE cells treated by different concentrations of AF was detected by ROS assay. (b) The expression of AMPK/mTOR in KLE cells treated by different concentrations of AF was analyzed by western blot. **P < 0.01 and ***P < 0.001 vs. Control group.

The mechanism that AF regulated ROS/AMPK/mTOR signaling pathway was analyzed by western blot. AF with different concentrations (50, 70, and 100 μM) gradually promoted ROS level in KLE cells (Figure 4a). The expression of p/t-AMPK was upregulated and p/t-mTOR was downregulated by AF in a concentration-dependent manner (Figure 4b).

### AF inhibited the proliferation and promoted the apoptosis of endometrial cancer cells by regulating ROS/AMPK/mTOR signaling pathway

**Figure 5.**
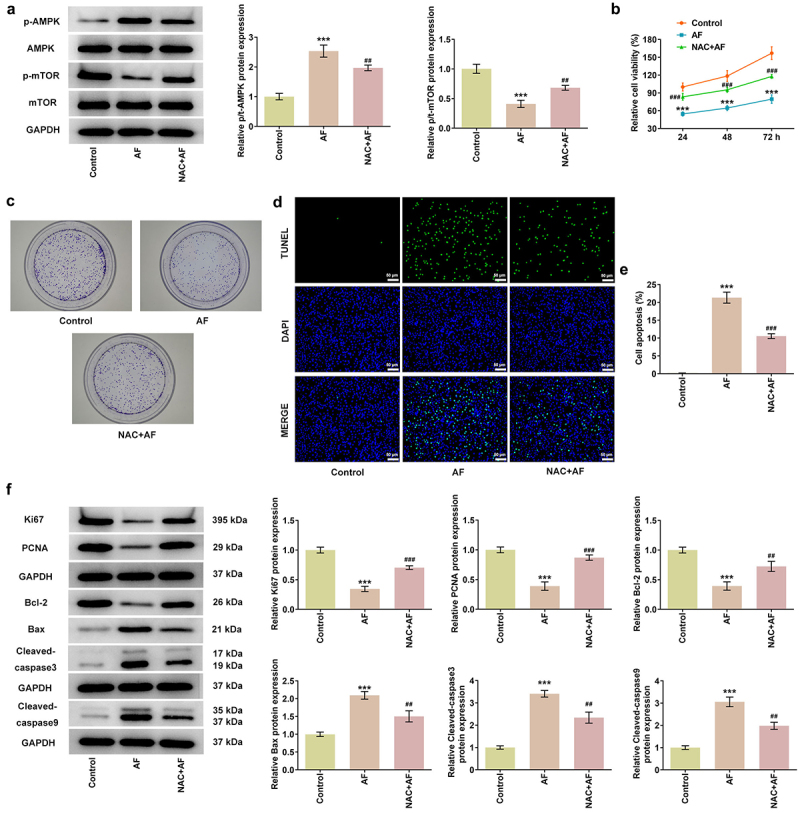
AF inhibited the proliferation and promoted the apoptosis of endometrial cancer cells by regulating ROS/AMPK/mTOR signaling pathway. (a) The expression of AMPK/mTOR in KLE cells treated by AF and NAC was analyzed by western blot. The viability (b) and proliferation (c) of KLE cells treated by AF and NAC were detected by CCK-8 and clone formation assays. (d and e) The apoptosis of KLE cells treated by AF and NAC was analyzed by TUNEL assay. (f) The expressions of proteins associated with proliferation and apoptosis in KLE cells treated by AF and NAC were determined by western blot. ***P < 0.001 vs. Control group. ^##^P < 0.01 and ^###^P < 0.001 vs. AF group.

NAC, which is an inhibitor of ROS, was used to confirm that AF regulated the proliferation and apoptosis of KLE cells through ROS/AMPK/mTOR signaling pathway. NAC treatment suppressed the expression of p/t-AMPK and promoted the expression of p/t-mTOR in AF-treated KLE cells (Figure 5a). NAC treatment improved the viability and proliferation (Figure 5b and c) but decreased the apoptosis (Figure 5d and e) of AF-treated KLE cells. The expressions of Ki67, PCNA, and Bcl-2 were risen and the expressions of Bax, cleaved-caspase3/caspase3 and cleaved-caspase9/caspase9 were declined in NAC+AF group compared with that in AF group (Figure 5f).

### AF promoted ferroptosis in endometrial cancer cells by regulating the ROS/AMPK/mTOR signaling pathway

**Figure 6.**
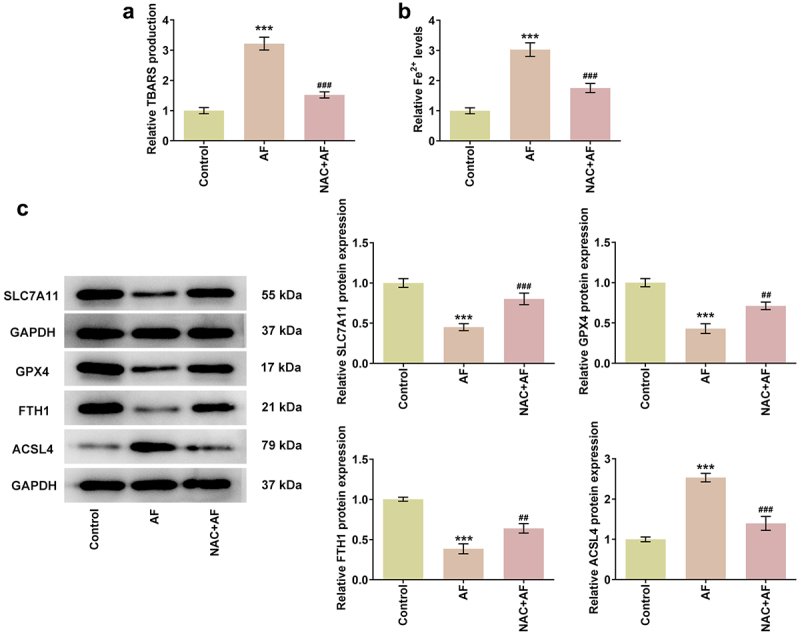
AF promoted ferroptosis in endometrial cancer cells by regulating the ROS/AMPK/mTOR signaling pathway. The TBARS production (a) and level of Fe^2+^ (b) in KLE cells treated by AF and NAC were detected by TBARS and Iron assays. (c) The expressions of proteins associated with ferroptosis in KLE cells treated by AF and NAC were determined by western blot. ***P < 0.001 vs. Control group. ^##^P < 0.01 and ^###^P < 0.001 vs. AF group.

NAC, which is an inhibitor of ROS, was used to confirm that AF regulated the ferroptosis of KLE cells through ROS/AMPK/mTOR signaling pathway. NAC treatment suppressed TBARS production and Fe^2+^ level in AF-treated KLE cells (Figure 6a and b). The expressions of ferroptosis-related proteins (SLC7A11, GPX4, FTH1, and ACSL4) were partially recovered in NAC+AF group compared with that in AF group (Figure 6c).

## Discussion

The prevention and treatment of tumor by traditional Chinese Medicine (TCM) are guided by the theory of traditional Chinese medicine and the use of natural medication orientation. TCM plays an important role in the intervention of precancerous lesions, improvement of mass of life, prevention of metastasis and recurrence, anti-tumor multidrug resistance, reduction of toxicity and increase of efficacy, and survival of tumor [19].

In recent years, more and more scholars have paid attention to the anticancer mechanism of AF. Many studies have shown that AF had anticancer effects on a variety of tumors. AF effectively suppressed cell growth and invasion and promoted G1 cell-cycle arrest and apoptosis of non-small cell lung cancer cells [20]. AF treatment inhibited the migration and invasion of TGF-β-induced A549 lung cancer cells and suppressed the metastasis of A549 lung cancer cells in mice [21]. AF promoted apoptosis and suppressed proliferation of breast cancer cells by inhibiting the fatty acid synthase [22]. AF enhanced apoptosis of cervical cancer cells by suppressing E7 expression and cell cycle arrest [23]. The present study indicated that AF could suppress the proliferation and promote the apoptosis of endometrial cancer cells.

Ferroptosis, as a regulatory cell death mode defined in recent years, is characterized by intracellular free iron increase and lipid peroxide accumulation [24]. With the deepening research of ferroptosis, the regulatory mechanism of ferroptosis in tumor had been improved, and more and more evidences showed that triggering ferroptosis in tumor cells was expected to become a new tumor treatment strategy. Erastin induced intracellular iron accumulation to promote ferroptosis of ovarian cancer cells [25]. In pancreatic ductal adenocarcinoma cells, artemisinin promoted ferroptosis by inducing the production of lipid ROS [26]. Exposure of ovarian cancer cells to high concentrations of artemisinin could cause ROS-dependent cell death, thus resulting in cell stagnation in the G2/M phase [27]. Juglone induced ferroptosis by Fe^2+^ accumulation, lipid peroxidation, and GSH depletion to inhibit cell migration and endoplasmic reticulum stress of endometrial carcinoma Ishikawa cells [18]. Our data confirmed that AF induced the ferroptosis of endometrial carcinoma KLE cells by promoting the production of TBAR, which indicated the lipid peroxidation level, and increasing the Fe^2+^ accumulation.

GPX4, a negative regulator of ferroptosis, could inhibit lipoxygenase-mediated lipid peroxidation by reducing phospholipid hydrogen peroxide, thus preventing ferroptosis [28,29]. Beclin1 could form a complex with SLC7A11 to inhibit system Xc^−^ activity, resulting in increased lipid peroxidation and enhanced ferroptosis induced by inducer [30]. ACSL4 could acetylate arachidonic acid, and the acetylated arachidonic acid could enter membrane phospholipid [31]. ROS could react with arachidonic acid on lipid membrane to induce lipid peroxidation and produce lipid ROS. Knockdown of ACSL4 inhibited erastin-induced ferroptosis [32]. FTH1, the functional subunit of the major iron storage protein ferritin, possessed ferroxidase activity and efficiently reduced the toxicity of Fe^2+^ [33]. Furthermore, FTH1 suppressed the ferroptosis of hepatocellular carcinoma cells [34] and leukemia cells [13]. In this study, after treatment of AF, the expressions of SLC7A11, GPX4, and FTH1 were upregulated, and the expression of ACSL4 was downregulated, indicating that AF induced the ferroptosis of endometrial carcinoma KLE cells.

The accumulation of lipid peroxides, especially phospholipid peroxides, is considered to be the landmark event of ferroptosis [35]. Lipid ROS accumulation is the main characteristic of ferroptosis [36]. SIRT3 knockdown suppressed AMPK-mTOR pathway and promoted the expression of GPX4, thereby suppressing autophagy and ferroptosis [37]. IMCA induced ferroptosis by decreasing SLC7A11 expression via the regulation of AMPK/mTOR pathway [38]. Bisphenol A induced ferroptosis of renal tubular epithelial cells by activating AMPK-mTOR-ULK1 pathway [39]. Here, we demonstrated that AF induced ferroptosis by activating ROS/AMPK/mTOR pathway, which was then reversed by NAC pretreatment.

## Conclusion

AF suppressed proliferation and promoted apoptosis as well as ferroptosis of endometrial carcinoma cells through the activation of ROS/AMPK/mTOR pathway. However, NAC could reverse the effects of AF on endometrial carcinoma cells. There are also some limitations of our study. According to Super-PRED (https://prediction.charite.de/index.php), we can see that AF may interact with ER. The effects of selective antagonists for ER on apoptosis and signaling pathways induced by AF will be explored in future.
